# Bis­(di­methylamine-κ*N*)bis[4-(1,2,4-triazol-1-yl)benzoato-κ*O*]copper(II)

**DOI:** 10.1107/S2414314622000463

**Published:** 2022-01-14

**Authors:** Lin Liu, Zheng-Bo Han

**Affiliations:** a College of Chemistry, Liaoning University, Shenyang, 110036, People’s Republic of China; Vienna University of Technology, Austria

**Keywords:** crystal structure, coordination polymer, hydrogen bond

## Abstract

In the title compound, [Cu(C_9_H_6_N_3_O_2_)_2_(C_2_H_7_N)_2_], the Cu^2+^ cation is situated on an inversion center and is coordinated by the N atoms of two di­methyl­amine ligands and the carboxyl­ate O atoms of two 4-(1,2,4-triazol-1-yl)benzoate anions, leading to a slightly distorted square-planar N_2_O_2_ coordination environment. In the crystal, inter­molecular N—H⋯N hydrogen bonds between the amine function and the central N atom of the triazole ring lead to the formation of ribbons parallel to [1  1]. Weak inter­molecular C—H⋯O hydrogen-bonding inter­actions are also observed that consolidate the crystal packing.

## Abstract 2

A three-dimensional supermolecule based on 4-(1,2,4-triazol-1-yl)benzoic acid was prepared under solvothermal conditions. In the crystal, inter­molecular N—H⋯N hydrogen bonds between the amine function and the central N atom of the triazole ring lead to the formation of ribbons parallel to [1 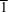 1].

## Graphical abstract

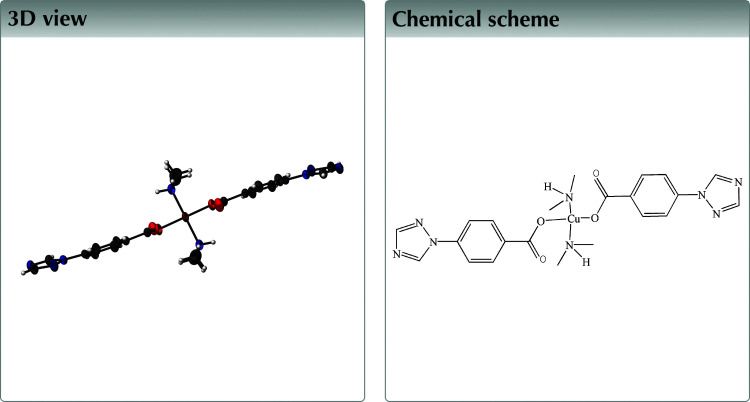

## Structure description

The rational design of coordination polymers is based on the combination of metal ions and versatile organic ligands, resulting in various supra­molecular assemblies. The resulting crystal structures determine the potential applications of the coordination polymers. Different polymers based on 4-(1,2,4-triazol-1-yl)benzoic acid complexes have been reported (Du *et al.*, 2014 ▸). They not only feature structural varieties, but also can be applied in gas storage (Wang *et al.*, 2012 ▸). In this context we have investigated crystals formed from a copper(II) solution and 4-(1,2,4-triazol-1-yl)benzoic acid under solvothermal conditions.

**Figure 1:**
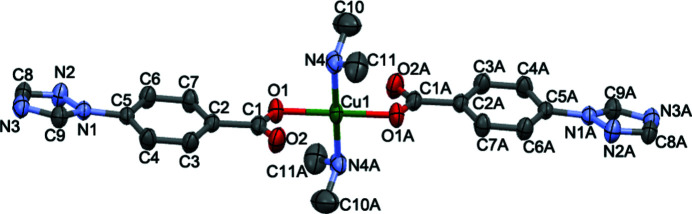
The mol­ecular structure of the title compound, with atom labelling and displacement ellipsoids drawn at the 30% probability level. H atoms have been omitted for clarity. [Symmetry code: (A) −*x* + 2, −*y*, −*z* + 1.]

**Figure 2:**
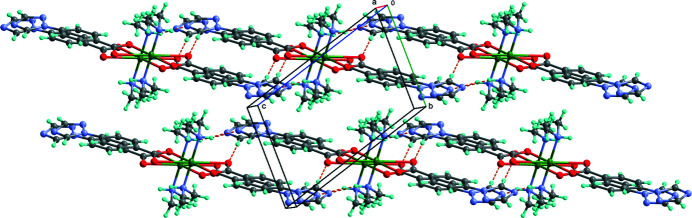
The crystal packing of the complex mol­ecules. Hydrogen-bonding inter­actions are shown as dashed lines.

As shown in Fig. 1 ▸, the asymmetric unit of the title compound comprises one Cu^II^ atom, one 4-(1,2,4-triazol-1-yl)benzoate ligand, and one di­methyl­amine mol­ecule generated *in situ* from the decomposition of the solvent dimethyl formamide. The complete mol­ecule is generated by inversion symmetry. The Cu^II^ atom has a distorted square-planar coordination environment, being coordinated by two symmetry-related benzoato O atoms [Cu—O1 = 1.9611 (14) Å] and two symmetry-related N atoms [Cu—N4 = 2.0096 (19) Å of the amine ligands. The second carboxyl­ate O atom of the anion seems to be too far away [Cu—O2 = 2.80136 (19) Å] to contribute to a significant bonding. Nevertheless, the non-bonding O2 atom is involved as an acceptor in weak C—H⋯O hydrogen-bonding inter­actions (Table 1 ▸, Fig. 2 ▸). Stronger N—H⋯N hydrogen bonds between the amine NH group and the central N atom of the triazole ring are also observed.

## Synthesis and crystallization

A mixture of Cu(NO_3_)_2_·3H_2_O (0.0725 mg, 0.3 mmol), 4-(1,2,4-triazol-1-yl)benzoic acid (0.057 g, 0.3 mmol), di­methyl­formamide (5 ml), ethanol (5 ml) and water (5 ml) was placed in a Teflon reactor with a 23 ml capacity, which was heated at 433 K for 3 days and then cooled to room temperature at a rate of 10 K h^−1^. Blue block-shaped crystals of the title compound were obtained in 52% yield after being washed with di­methyl­formamide and dried in air.

## Refinement

Crystal data, data collection and structure refinement details are summarized in Table 2 ▸.

## Supplementary Material

Crystal structure: contains datablock(s) I. DOI: 10.1107/S2414314622000463/wm4159sup1.cif

Structure factors: contains datablock(s) I. DOI: 10.1107/S2414314622000463/wm4159Isup2.hkl

CCDC reference: 2141669

Additional supporting information:  crystallographic information; 3D view; checkCIF report

## Figures and Tables

**Table 1:** Hydrogen-bond geometry (Å, °)

*D*—H⋯*A*	*D*—H	H⋯*A*	*D*⋯*A*	*D*—H⋯*A*
N4—H4⋯N3^i^	0.91	2.17	3.034 (3)	160
C9—H9*A*⋯O2^ii^	0.93	2.50	3.428 (3)	173

Symmetry codes: (i) 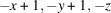 ; (ii) 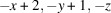 .

**Table 2:** Experimental details

Crystal data	
Chemical formula	[Cu(C_9_H_6_N_3_O_2_)_2_(C_2_H_7_N)_2_]
*M*_r_	530.06
Crystal system, space group	Triclinic, *P* 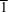
Temperature (K)	293
*a*, *b*, *c* (Å)	6.3657 (5), 8.1428 (7), 12.1896 (11)
α, β, γ (°)	72.595 (2), 89.376 (2), 87.805 (2)
*V* (Å^3^)	602.47 (9)
*Z*	1
Radiation type	Mo *K*α
μ (mm^−1^)	0.95
Crystal size (mm)	0.36 × 0.32 × 0.27

Data collection	
Diffractometer	Bruker SMART CCD
Absorption correction	Multi-scan (*SADABS*; Bruker, 2002 ▸)
*T*_min_, *T*_max_	0.727, 0.785
No. of measured, independent and observed [*I* > 2σ(*I*)] reflections	3971, 2727, 2345
*R*_int_	0.017
(sin θ/λ)_max_ (Å^−1^)	0.650

Refinement	
*R*[*F* ^2^ > 2σ(*F* ^2^)], *wR*(*F* ^2^), *S*	0.038, 0.093, 1.03
No. of reflections	2727
No. of parameters	160
H-atom treatment	H-atom parameters constrained
Δρ_max_, Δρ_min_ (e Å^−3^)	0.28, −0.20

Computer programs: *SMART* and *SAINT* (Bruker, 2002 ▸), *SHELXS97* (Sheldrick, 2008 ▸), *SHELXL* (Sheldrick, 2015 ▸), *Mercury* (Macrae *et al.*, 2020 ▸) and *publCIF* (Westrip, 2010 ▸).

## full crystallographic data

### Crystal data

**Table:**

[Cu(C_9_H_6_N_3_O_2_)_2_(C_2_H_7_N)_2_]	*Z* = 1
*M**_r_* = 530.06	*F*(000) = 275
Triclinic, *P*1	*D*_x_ = 1.461 Mg m^−^^3^
*a* = 6.3657 (5) Å	Mo *K*α radiation, λ = 0.71073 Å
*b* = 8.1428 (7) Å	Cell parameters from 1477 reflections
*c* = 12.1896 (11) Å	θ = 2.6–26.4°
α = 72.595 (2)°	µ = 0.95 mm^−^^1^
β = 89.376 (2)°	*T* = 293 K
γ = 87.805 (2)°	Block, blue
*V* = 602.47 (9) Å^3^	0.36 × 0.32 × 0.27 mm

### Data collection

**Table:**

Bruker SMART CCD diffractometer	2727 independent reflections
Radiation source: fine-focus sealed tube	2345 reflections with *I* > 2σ(*I*)
Detector resolution: 10.0 pixels mm^-1^	*R*_int_ = 0.017
ω scan	θ_max_ = 27.5°, θ_min_ = 2.6°
Absorption correction: multi-scan (*SADABS*; Bruker, 2002)	*h* = −8→8
*T*_min_ = 0.727, *T*_max_ = 0.785	*k* = −10→10
3971 measured reflections	*l* = −10→15

### Refinement

**Table:**

Refinement on *F*^2^	0 restraints
Least-squares matrix: full	Hydrogen site location: mixed
*R*[*F*^2^ > 2σ(*F*^2^)] = 0.038	H-atom parameters constrained
*wR*(*F*^2^) = 0.093	*w* = 1/[σ^2^(*F*_o_^2^) + (0.0482*P*)^2^ + 0.1332*P*] where *P* = (*F*_o_^2^ + 2*F*_c_^2^)/3
*S* = 1.03	(Δ/σ)_max_ < 0.001
2727 reflections	Δρ_max_ = 0.28 e Å^−^^3^
160 parameters	Δρ_min_ = −0.20 e Å^−^^3^

### Special details

**Table:**

Geometry. All esds (except the esd in the dihedral angle between two l.s. planes) are estimated using the full covariance matrix. The cell esds are taken into account individually in the estimation of esds in distances, angles and torsion angles; correlations between esds in cell parameters are only used when they are defined by crystal symmetry. An approximate (isotropic) treatment of cell esds is used for estimating esds involving l.s. planes.

### Fractional atomic coordinates and isotropic or equivalent isotropic displacement parameters (Å^2^)

**Table:**

	*x*	*y*	*z*	*U*_iso_*/*U*_eq_	
Cu1	1.000000	0.000000	0.500000	0.03668 (13)	
O1	0.8278 (3)	0.19597 (19)	0.40688 (13)	0.0456 (4)	
N4	0.8929 (3)	−0.1440 (2)	0.40592 (16)	0.0478 (5)	
H4	0.834029	−0.069234	0.342060	0.072*	
O2	1.0899 (3)	0.2218 (2)	0.28158 (16)	0.0564 (5)	
N3	0.3345 (3)	0.9752 (2)	−0.18059 (16)	0.0470 (5)	
C8	0.1967 (4)	0.9638 (3)	−0.0944 (2)	0.0475 (5)	
H8A	0.069359	1.026329	−0.105518	0.057*	
N1	0.4455 (3)	0.7973 (2)	−0.01740 (15)	0.0357 (4)	
C1	0.9149 (3)	0.2671 (3)	0.31047 (18)	0.0397 (5)	
C2	0.7914 (3)	0.4135 (2)	0.22845 (17)	0.0347 (4)	
C3	0.8841 (3)	0.5111 (3)	0.1285 (2)	0.0443 (5)	
H3A	1.024977	0.489576	0.114867	0.053*	
C4	0.7728 (3)	0.6395 (3)	0.0487 (2)	0.0462 (5)	
H4A	0.838250	0.704495	−0.017864	0.055*	
C5	0.5630 (3)	0.6712 (2)	0.06815 (17)	0.0332 (4)	
C6	0.4692 (3)	0.5793 (3)	0.16901 (19)	0.0429 (5)	
H6A	0.329651	0.603747	0.183663	0.052*	
C7	0.5831 (3)	0.4506 (3)	0.24845 (18)	0.0438 (5)	
H7A	0.518902	0.388216	0.316208	0.053*	
C9	0.4888 (4)	0.8692 (3)	−0.12927 (18)	0.0437 (5)	
H9A	0.611277	0.846901	−0.165463	0.052*	
N2	0.2529 (3)	0.8588 (2)	0.00657 (16)	0.0467 (5)	
C11	1.0630 (5)	−0.2323 (4)	0.3597 (3)	0.0766 (9)	
H11A	1.164757	−0.150450	0.321505	0.115*	
H11B	1.129585	−0.320275	0.421504	0.115*	
H11C	1.005285	−0.283743	0.305845	0.115*	
C10	0.7338 (6)	−0.2657 (4)	0.4651 (3)	0.0853 (10)	
H10A	0.624985	−0.205376	0.494376	0.128*	
H10B	0.674023	−0.317305	0.411925	0.128*	
H10C	0.798323	−0.353837	0.527585	0.128*	

### Atomic displacement parameters (Å^2^)

**Table:**

	*U*^11^	*U*^22^	*U*^33^	*U*^12^	*U*^13^	*U*^23^
Cu1	0.0456 (2)	0.0340 (2)	0.02439 (18)	0.00871 (14)	−0.00500 (14)	−0.00062 (13)
O1	0.0569 (9)	0.0417 (8)	0.0312 (7)	0.0137 (7)	−0.0080 (7)	−0.0019 (6)
N4	0.0643 (12)	0.0411 (10)	0.0328 (9)	0.0055 (9)	−0.0129 (9)	−0.0034 (8)
O2	0.0430 (9)	0.0580 (10)	0.0580 (11)	0.0168 (8)	−0.0055 (8)	−0.0038 (8)
N3	0.0571 (11)	0.0418 (10)	0.0366 (10)	0.0088 (8)	−0.0123 (9)	−0.0043 (8)
C8	0.0512 (13)	0.0448 (12)	0.0427 (12)	0.0162 (10)	−0.0140 (10)	−0.0089 (10)
N1	0.0387 (9)	0.0317 (8)	0.0330 (8)	0.0042 (7)	−0.0060 (7)	−0.0045 (7)
C1	0.0453 (12)	0.0350 (10)	0.0360 (11)	0.0061 (9)	−0.0121 (9)	−0.0069 (8)
C2	0.0388 (10)	0.0325 (9)	0.0305 (10)	0.0036 (8)	−0.0075 (8)	−0.0060 (8)
C3	0.0333 (10)	0.0467 (12)	0.0446 (12)	0.0055 (9)	−0.0017 (9)	−0.0018 (10)
C4	0.0398 (11)	0.0451 (12)	0.0412 (12)	0.0019 (9)	0.0036 (9)	0.0055 (9)
C5	0.0365 (10)	0.0288 (9)	0.0315 (10)	0.0025 (8)	−0.0064 (8)	−0.0051 (7)
C6	0.0350 (10)	0.0496 (12)	0.0366 (11)	0.0087 (9)	0.0009 (9)	−0.0027 (9)
C7	0.0449 (12)	0.0460 (12)	0.0311 (10)	0.0067 (9)	0.0029 (9)	0.0017 (9)
C9	0.0482 (12)	0.0427 (11)	0.0349 (11)	0.0050 (9)	−0.0043 (9)	−0.0040 (9)
N2	0.0425 (10)	0.0498 (11)	0.0407 (10)	0.0145 (8)	−0.0048 (8)	−0.0049 (8)
C11	0.100 (2)	0.0749 (19)	0.0628 (18)	0.0288 (17)	−0.0177 (17)	−0.0357 (16)
C10	0.103 (3)	0.082 (2)	0.065 (2)	−0.0332 (19)	−0.0135 (18)	−0.0088 (17)

### Geometric parameters (Å, º)

**Table:**

Cu1—O1	1.9611 (14)	C2—C3	1.380 (3)
Cu1—O1^i^	1.9612 (14)	C2—C7	1.384 (3)
Cu1—N4	2.0096 (19)	C3—C4	1.375 (3)
Cu1—N4^i^	2.0096 (19)	C3—H3A	0.9300
O1—C1	1.276 (3)	C4—C5	1.382 (3)
N4—C10	1.470 (4)	C4—H4A	0.9300
N4—C11	1.475 (4)	C5—C6	1.377 (3)
N4—H4	0.9071	C6—C7	1.382 (3)
O2—C1	1.240 (3)	C6—H6A	0.9300
N3—C9	1.314 (3)	C7—H7A	0.9300
N3—C8	1.345 (3)	C9—H9A	0.9300
C8—N2	1.315 (3)	C11—H11A	0.9600
C8—H8A	0.9300	C11—H11B	0.9600
N1—C9	1.343 (3)	C11—H11C	0.9600
N1—N2	1.368 (2)	C10—H10A	0.9600
N1—C5	1.421 (2)	C10—H10B	0.9600
C1—C2	1.507 (3)	C10—H10C	0.9600
			
O1—Cu1—O1^i^	180.00 (8)	C3—C4—C5	119.5 (2)
O1—Cu1—N4	89.16 (7)	C3—C4—H4A	120.2
O1^i^—Cu1—N4	90.84 (7)	C5—C4—H4A	120.2
O1—Cu1—N4^i^	90.84 (7)	C6—C5—C4	119.96 (18)
O1^i^—Cu1—N4^i^	89.16 (7)	C6—C5—N1	120.69 (18)
N4—Cu1—N4^i^	180.00 (7)	C4—C5—N1	119.34 (18)
C1—O1—Cu1	111.32 (13)	C5—C6—C7	119.81 (19)
C10—N4—C11	111.0 (2)	C5—C6—H6A	120.1
C10—N4—Cu1	113.77 (18)	C7—C6—H6A	120.1
C11—N4—Cu1	112.97 (17)	C6—C7—C2	120.9 (2)
C10—N4—H4	108.7	C6—C7—H7A	119.6
C11—N4—H4	103.5	C2—C7—H7A	119.6
Cu1—N4—H4	106.2	N3—C9—N1	110.8 (2)
C9—N3—C8	102.46 (18)	N3—C9—H9A	124.6
N2—C8—N3	116.0 (2)	N1—C9—H9A	124.6
N2—C8—H8A	122.0	C8—N2—N1	101.72 (18)
N3—C8—H8A	122.0	N4—C11—H11A	109.5
C9—N1—N2	109.03 (17)	N4—C11—H11B	109.5
C9—N1—C5	129.63 (18)	H11A—C11—H11B	109.5
N2—N1—C5	121.27 (17)	N4—C11—H11C	109.5
O2—C1—O1	124.09 (19)	H11A—C11—H11C	109.5
O2—C1—C2	119.7 (2)	H11B—C11—H11C	109.5
O1—C1—C2	116.21 (18)	N4—C10—H10A	109.5
C3—C2—C7	118.30 (18)	N4—C10—H10B	109.5
C3—C2—C1	119.88 (19)	H10A—C10—H10B	109.5
C7—C2—C1	121.79 (19)	N4—C10—H10C	109.5
C4—C3—C2	121.5 (2)	H10A—C10—H10C	109.5
C4—C3—H3A	119.3	H10B—C10—H10C	109.5
C2—C3—H3A	119.3		
			
C9—N3—C8—N2	−0.1 (3)	C9—N1—C5—C4	17.1 (3)
Cu1—O1—C1—O2	−2.6 (3)	N2—N1—C5—C4	−166.2 (2)
Cu1—O1—C1—C2	176.67 (13)	C4—C5—C6—C7	−2.7 (3)
O2—C1—C2—C3	−8.7 (3)	N1—C5—C6—C7	176.47 (19)
O1—C1—C2—C3	172.0 (2)	C5—C6—C7—C2	0.5 (4)
O2—C1—C2—C7	169.3 (2)	C3—C2—C7—C6	1.6 (3)
O1—C1—C2—C7	−10.0 (3)	C1—C2—C7—C6	−176.4 (2)
C7—C2—C3—C4	−1.6 (4)	C8—N3—C9—N1	0.0 (3)
C1—C2—C3—C4	176.4 (2)	N2—N1—C9—N3	0.0 (3)
C2—C3—C4—C5	−0.5 (4)	C5—N1—C9—N3	177.02 (19)
C3—C4—C5—C6	2.7 (3)	N3—C8—N2—N1	0.1 (3)
C3—C4—C5—N1	−176.5 (2)	C9—N1—N2—C8	0.0 (2)
C9—N1—C5—C6	−162.0 (2)	C5—N1—N2—C8	−177.35 (18)
N2—N1—C5—C6	14.7 (3)		

Symmetry code: (i) −*x*+2, −*y*, −*z*+1.

### Hydrogen-bond geometry (Å, º)

**Table:**

*D*—H···*A*	*D*—H	H···*A*	*D*···*A*	*D*—H···*A*
N4—H4···N3^ii^	0.91	2.17	3.034 (3)	160
C9—H9*A*···O2^iii^	0.93	2.50	3.428 (3)	173

Symmetry codes: (ii) −*x*+1, −*y*+1, −*z*; (iii) −*x*+2, −*y*+1, −*z*.
